# Protective Effect of the Total Triterpenes of* Euscaphis konishii* Hayata Pericarp on Bacillus Calmette-Guérin Plus Lipopolysaccharide-Induced Liver Injury

**DOI:** 10.1155/2019/1806021

**Published:** 2019-04-04

**Authors:** Wei Huang, Hui Ding, Lu-yao Chen, Lin Ni, Yi-fang Ruan, Xiao-xing Zou, Min Ye, Shuang-quan Zou

**Affiliations:** ^1^College of Life Sciences, Fujian Agriculture and Forestry University, Fuzhou 350002, China; ^2^Engineering Research Institute of Conservation, Utilization of Natural Bioresources, Fujian Agriculture and Forestry University, Fuzhou 350002, China; ^3^Forestry College, Fujian Agriculture and Forestry University, Fuzhou 350002, China; ^4^College of Plant Protection, Fujian Agriculture and Forestry University, Fuzhou 350002, China; ^5^Fujian Key Laboratory of Natural Medicine Pharmacology, School of Pharmacy, Fujian Medical University, Fuzhou 350004, China

## Abstract

**Background:**

Liver injury has been recognized as a primary cause of hepatic morbidity and mortality.* Euscaphis konishii* Hayata, also called* Euscaphis fukienensis* Hsu, is usually used as a detumescent and analgesic agent to improve liver function in South China, but its mechanism of action and chemical composition are unclear.

**Objective:**

The main aim of the study was to investigate the constituent and potential hepatoprotective mechanism of the total triterpenes of* E. konishii* pericarp (TTEP).

**Methods:**

The constituent of TTEP was analyzed by a series of silica gel column to get single compounds and then identified by NMR and MS.* In vitro* assays were conducted to test the free radical scavenging activity of TTEP. The BCG/LPS-induced immunological livery injury mice model was established to clarify the hepatoprotective effect of TTEP* in vivo*.

**Results:**

8 pentacyclic triterpene acids were separated and identified by NMR and MS. TTEP treatment (50, 100, and 200 mg/Kg) improved the immune function of the BCG/LPS-infected mice, dose-dependently alleviated the BCG/LPS-induced inflammation and oxidative stress, and ameliorated the hepatocyte apoptosis in the liver tissue.

**Conclusion:**

The pericarp of* E. konishii* may be further considered as a potent natural food for liver disease treatment.

## 1. Introduction

Liver, the central organ of metabolism, digestion, excretion, detoxification, and immunity, is susceptible to a variety of factors including viral infections, alcohol, drug abuse, and autoimmune attack of hepatocytes [1, 2]. Viral liver injury, which can progress into hepatitis, cirrhosis, and even hepatocellular carcinoma without effective therapies at present, is now becoming one of the predominant causes of human hepatic morbidity and mortality worldwide [3]. The endotoxin/lipopolysaccharide (LPS) induced liver injury has been recognized as the pathological basis of viral hepatic diseases [4], and the mice induced by LPS pretreated by Bacillus Calmette-Guérin (BCG) has been accepted as a classical experimental model to study the clinically viral fulminant hepatic failure, which is characterized by immune dysfunction, oxidative stress, inflammation, and apoptosis [5–7].

It has been clarified that BCG priming phase modulates the proliferation and differentiation of immune cells such as CD3^+^, CD4^+^, and CD8^+^ T lymphocytes and induce them infiltration into the liver lobules to cause injury [8]. The subsequent LPS injection elicits dramatic hepatic damage, associated with the release of various proinflammatory chemokines, cytokines, and reactive oxygen/nitrogen species (ROS/RNS) [9–11]. Liver injury induced by LPS activates the Toll-like receptor 4 (TLR4)/NF-*κ*B signaling, promoting the expression of many inflammatory cytokines and chemokines, including Interleukin-6 (IL-6), tumor necrosis factor-*α* (TNF-*α*), and nitric oxide (NO) [12, 13]. The imbalance of endogenous enzymes including glutathione (GSH), superoxide dismutase (SOD), and catalase (CAT) is an important pathogenesis of liver diseases, which could be reflected by aberrant expression of antioxidant signaling such as Nrf2/HO-1 pathway [14, 15]. Moreover, excessive oxidative stress and inflammatory mediators are able to trigger the mitogen-activated protein kinases (MAPK) pathway, resulting in the activation of apoptotic signaling [16, 17].

*Euscaphis konishii* Hayata, also called* Euscaphis fukienensis* Hsu, is an evergreen indeciduous small arbor that is peculiarly growing in the South of China [18–20]. The roots, branches, leaves, flowers, and fruits have been used as traditional Chinese medicine with detumescence, analgesia, and antirheumatic properties [21, 22]. Phytochemical investigations showed that the main components of the pericarp are triterpenes, flavonoids, and polyphenols, and the total triterpenes accounted for about 10% of the ethanol extract [23]. The aim of the present work is to analyze the constituent of the total triterpene of* E. konishii* pericarp (TTEP) and investigate the hepatoprotective activity of TTEP using the BCG/LPS-induced mice model. In the present work, we found that TTEP possessed strong free radicals scavenging activity* in vitro* and 8 major pentacyclic triterpene acids were identified by NMR and MS. With oral administration, TTEP improved the immune function of BCG/LPS-infected mice, alleviated inflammation, attenuated oxidative stress, and suppressed apoptosis in the BCG/LPS-infected mice liver. Therefore, the pericarp of* E. konishii* should be further considered as a potent natural food for immunological liver disease treatment.

## 2. Materials and Methods

### 2.1. Reagents

Mycobacterium tuberculosis Bacillus Calmette-Guérin (BCG) vaccine was purchased from Shanghai Institute of Biological Product (China). Lipopolysaccharide (LPS) was obtained from Solarbio (Beijing, China). Bifendate was got from Beijing Union Pharm. PerCP Hamster Anti-Mouse CD3e (No. 553067), APC Rat Anti-Mouse CD4 (No. 553051), and PE Rat Anti-Mouse CD8a (No. 553032) were obtained from BD Biosciences. The kits for determining malondialdehyde (MDA) content, aspartate aminotransferase (AST), superoxide dismutase (SOD), alanine aminotransferase (ALT), glutathione peroxidase (GSH-Px), alkaline phosphatase (ALP), and catalase (CAT) activities were obtained from Jiancheng (Nanjing, China). The tumor necrosis factor-*α* (TNF-*α*) and interleukin-6 (IL-6) ELISA kits were purchased from the Enzyme-linked Biotechnology Co., Ltd. (Shanghai, China). Antibodies against Cleaved caspase-3, Cleaved caspase 9, NAD (P)H Dehydrogenase Quinone 1 (NQO1), and Cytochrome C (Cyt C) were obtained from Abcam. Antibodies against extracellular regulated protein kinase (ERK), I*κ*B-*β*, Phospho-ERK, Phospho-NF-*κ*B p65, Toll-like receptor (TLR4), p38, NF-*κ*B p65, Phospho-p38, and Phospho-I*κ*B-*β* were purchased from Cell Signaling Technology. Primary antibodies against Nuclear factor (erythroid-derived 2)-like 2 (Nrf2), c-Jun N-terminal kinase (JNK), Heme Oxygenase 1 (HO-1), Phospho-JNK, superoxide dismutase (SOD), Cytochrome c oxidase subunit 4 (COX4), Histone, Bcl-2, *β*-actin and Bax were got from Santa Cruz.

### 2.2. Plant Materials and Preparation

*Euscaphis Konishii *Hayata, identified by Prof. Shuang-quan Zou, was cultivated in Tiancheng Rock, Shaowu, Fujian province, China. Dried and powdered* E. Konishii* pericarp (12.0Kg) was extracted with 70% ethanol and concentrated to obtain ethanol extract (2.7 Kg). The ethanol extract was then eluted by silica gel column with different ratio of petroleum ether/ethyl acetate, petroleum ether/acetone, and chloroform/methanol. Each fraction was analyzed by the Liebermann-Burchard reagent (acetic anhydride-concentrated H_2_SO_4_) and spotted on TLC plates by spray reagent anisalehyde-H_2_SO_4_ to analyze the presence of triterpenes. The fractions that passed the triterpene tests were collected and enriched by vacuum evaporation to get the total triterpene of* E. Konishii* pericarp (TTEP) (282 g). The TTEP residue was then further separated by silica gel and Sephadex LH-20 column chromatography to get single compounds. Their structures were elucidated by 1D, 2D-NMR (BRUKER AV500-III, Switzerland) and MS (Waters ACQUITY QDa, USA).

### 2.3. *In Vitro* Free Radical Scavenging Activity of TTEP

#### 2.3.1. ABTS^+^ Scavenging Assay

The ABTS^+^ scavenging activity of TTEP was assayed according to the previously described method [24]. Vitamin C was used as positive control. Percentage inhibition of ABTS^+^ scavenging activity (%) = [(OD_control_- OD_sample_)/ OD_control_)] ×100, where OD_control_ is absorbance of ABTS radical and OD_sample_ is the absorbance of ABTS radical along with different concentrations of TTEP/positive control.

#### 2.3.2. DPPH Scavenging Assay

The DPPH scavenging activity of TTEP was assayed according to the previously described method [25]. Percentage inhibition of DPPH scavenging activity (%) = [(OD_control_- OD_sample_)/ OD_control_)]×100, where OD_control_ is absorbance of DPPH radical and OD_sample_ is the absorbance of DPPH radical along with different concentrations of TTEP/positive control.

#### 2.3.3. O_2_^−^ Scavenging Assay

The O_2_^−^ radical scavenging activity was measured by NADH-PMS-NBT system [26]. Vitamin C was used as positive control. Percentage inhibition of O_2_^−^ scavenging activity (%) = [(OD_control_- OD_sample_)/ OD_control_)]×100, where OD_control_ is absorbance of O_2_^−^ radical and OD_sample_ is the absorbance of O_2_^−^ radical along with different concentrations of TTEP/positive control.

#### 2.3.4. NO Radical Scavenging Assay

NO radical scavenging activity was performed according to previously described method [27]. Dexamethasone was used as positive control. Percentage inhibition of NO radical scavenging activity (%) = [(OD_control_- OD_sample_)/ OD_control_)]×100, where OD_control_ is absorbance of NO radical and OD_sample_ is the absorbance of NO radical along with different concentrations of TTEP/positive control.

### 2.4. Animal Experiments

Male BALB/c mice, 8-10 weeks old, and weighing 20-24 g were maintained under standard laboratory conditions. All animal experiments were supervised and approved by the Ethics Review committee for Animal Experimentation of Fujian Medical University, China.

#### 2.4.1. Evaluation of Acute Toxicity

TTEP was suspended in 0.5% sodium carboxymethylcellulose (CMC) solution and administered orally to mice at a dose of 10 mL/kg body weight. The toxicity of TTEP was examined at a dose of 1.0 g/Kg, which is equal to about 50.0 g* E. Konishii *pericarp /Kg. The mice were orally administered twice a day with 12 h interval and lasted for 3 days and then kept to observe in the coming two weeks. All mice were executed, and no histological abnormalities were noticed in the liver, heart, lung, and kidneys at the end of drug treatment.

#### 2.4.2. Establishment of the BCG/LPS-Induced Hepatic Injury Model and Drug Treatment

To assess the effect of TTEP on BCG/LPS-induced hepatic injury, mice were randomly divided into six groups (n=10). (I) Normal control group: mice were administered with the vehicle CMC saline solution for 14 days. (II) BCG/LPS model group: mice were treated with the vehicle for 14 days after injection via the tail vein with 125 mg/kg dose of BCG saline (approximately 5×10^7^ viable units per mouse). (III) BCG/LPS with Bifendate: mice were treated with BCG as model group and 200 mg/kg Bifendate for 14 days as positive control. (IV) 50 mg/kg TTEP: mice were injected with BCG as model group and administered with 50 mg/kg TTEP for 14 days. (V) 100 mg/kg TTEP: mice were treated with BCG as model group and 100 mg/kg TTEP for 14 days. (VI) 200 mg/kg TTEP: mice were treated with BCG as model group and 200 mg/kg TTEP for 14 days. The mice were then injected with LPS saline (125 *μ*g/kg) intravenously 2 h after the last dosage. Blood samples were collected in anticoagulant tubes 8 h after the LPS injection. Mice were then sacrificed, and thymus, liver, and spleen tissues were removed for further examination.

### 2.5. Measurement of T Lymphocyte Subgroups

20 *μ*L anticoagulant blood was mixed with 180 *μ*L erythrocyte lysate and placed stably for 10 min. Then 2 mL phosphate buffer was added to the above solution before centrifugation at 600 rpm for 5 min. The white blood cells were resuspended with phosphate buffer and incubated with CD3^+^, CD4^+^ and CD8^+^ antibody (2:1:1) for 15 min and analyzed with flow cytometry (BD FACSCalibur, USA)

### 2.6. Biochemical Determinations

#### 2.6.1. Measurement of Serum Enzyme Levels

The left mice blood sample were centrifuged to get the serum, and alanine aminotransferase (ALT), aspartate transaminase (AST), and alkaline phosphatase (ALP) activities were detected by colorimetric method by the commercial kits at automatic biochemical analyzer (TBA-120FR, Toshiba).

#### 2.6.2. Measurement of Anti-Inflammatory Biomarkers

The mice liver samples were homogenized with the icy saline and centrifuged at 3000 rpm at 4°C for 20 min. The supernatants were used to determine NO, TNF-*α*, and IL-6 with commercial kits following the instructions.

#### 2.6.3. Measurement of Antioxidant System

The liver homogenate was used to determine the concentrations of SOD, GSH, CAT, and malondialdehyde (MDA). The biomarkers were evaluated using diagnostic kits following the manufacturer's instructions.

### 2.7. Histophathology

The liver tissues were fixed with formalin, embedded with paraffin, and mounted on slides for hematoxylin and eosin (H&E) staining. Samples were analyzed using an inverted microscope (Olympus 1X73, Japan).

### 2.8. TUNEL Staining

Apoptotic hepatocytes were detected with TUNEL cell apoptosis detection kit (Biotin tagged POD method, KeyGEN BioTECH Corp., Ltd., Jiangsu, China) in paraffin-embedded section following the manufacturer's instructions.

### 2.9. Cytosolic and Nuclear Protein Fractionation

The liver tissue (200-300 mg) was ground on liquid nitrogen and homogenized in icy lysis buffer as described in [28]. The homogenates were centrifuged at 12,000 rpm to get the supernatant as the cytoplasmic fraction. The pellet was resuspended in nuclear extraction buffer for 40 min and centrifuged at 12,000 rpm for 15 min to get the supernatant as the nuclear fraction.

### 2.10. Isolation of Mitochondria from Liver Tissue

Liver tissue was homogenized with mitochondrial isolation buffer described in [29] to get a 10% (w/v) live homogenate and centrifuged at 600 g for 10 min to remove the tissue debris. The supernatant was further centrifuged at 10,000 g for 10 min to collect the mitochondrial rich fraction. The suspension was washed twice using the wash buffer to get the purified mitochondria.

### 2.11. Quantitative Real-Time PCR

Total RNA of liver tissue was extracted to synthesize the first strand cDNA, and quantitative RT-PCR analysis was performed in triplicate with StepOnePlus Real-Time PCR system (Life Technologies). Primer sequences were synthesized by Shanghai Generay Biotech Co., LTD (shown in Table 1) and the ΔΔCT method was adopted to calculate relative mRNA expression.

### 2.12. Western Blot

Liver tissue was homogenated with NP-40 lysis buffer (Beyotime Biotechnology, China) at 4°C and centrifuged at 12,000 ×* g* for 10 min to get the supernatant. Proteins were analyzed with SDS-PAGE, transferred to PVDF membranes, blotted with specific primary antibodies, and detected via incubation with horseradish peroxidase-conjugated secondary antibodies at the FluorChem Q (ProteinSimple).

### 2.13. Statistical Analysis

Data were represented as means ± standard deviation. Variance analysis was adopted for comparisons across multiple groups. PASW statistics 18 (SPSS) was used for statistical analysis, and* P* < 0.05 was considered to have statistical significance.

## 3. Results

### 3.1. Chemical Constituent of TTEP

**Figure 1:**
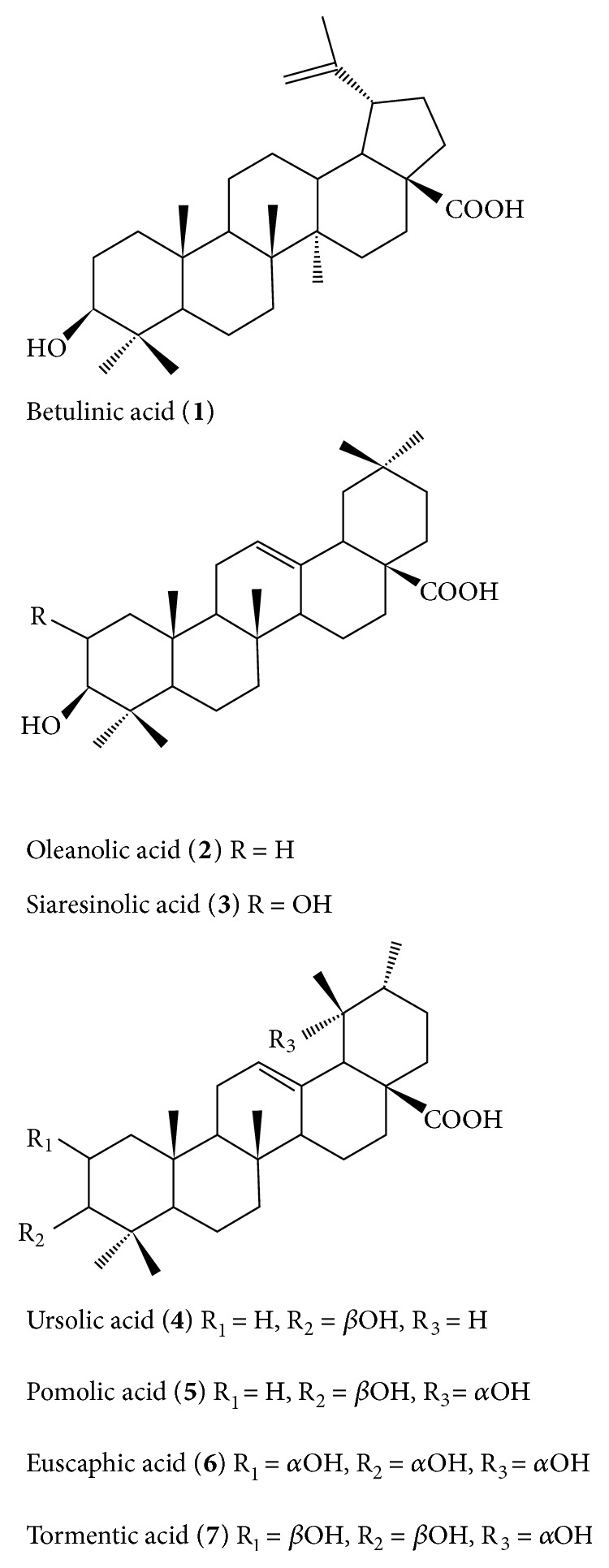
*Structures of isolated triterpenes from total triterpenes of Euscaphis Konishii Hayata pericarp (TTEP)*. TTEP was extracted by silica column gel and analyzed by Liebermann-Burchard reagent from* E. Konishii *pericarp ethanol extract. 8 single triterpenes were further separated and identified by NMR and MS.

To elucidate the chemical constituent of the total triterpenes of* E. konishii* pericarp (TTEP), we performed a series of silica gel column to obtain single triterpene compounds. Eight major triterpenes of* E. konishii* pericarp were separated and identified (shown in Figure 1): Betulinic acid (**1**, 8.0%), Oleanolic acid (**2**, 5.7%), Siaresinolic acid (**3**, 3.1%), Ursolic acid (**4**, 16.4 %), Pomolic acid (**5**, 9.8%), Euscaphic acid (**6**, 15.2%), Tormentic acid (**7**, 13.5%), and Corosolic acid (**8**, 6.8%) according to their NMR and MS data (shown in Figure 1 and Supplementary Materials (available here)).

### 3.2. *In Vitro* Free Scavenging Activity of TTEP

**Figure 2:**
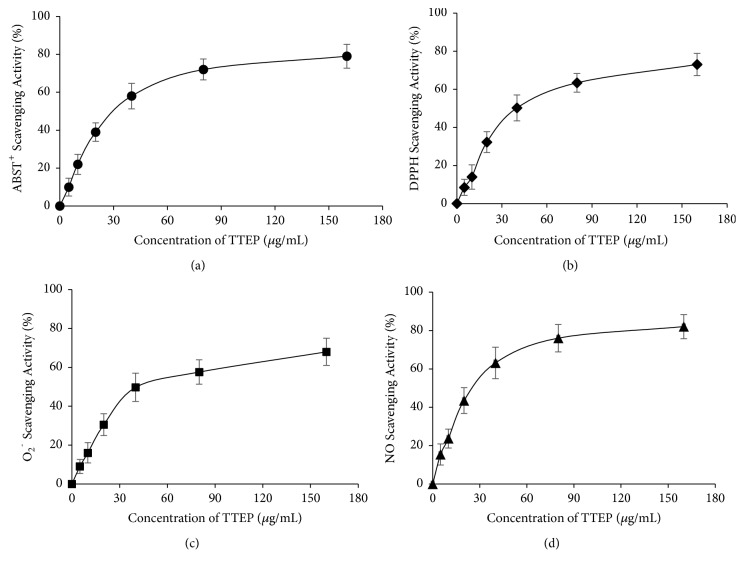
*In vitro antioxidant activity of TTEP*. Different concentrations of TTEP (5, 10, 20, 40, 80 and 160 *μ*g/mL) or vehicle were tested for the scavenging activity (mean ± SD, n = 3) on (a) ABTS+, (b) DPPH, (c) O_2_^−^, and (d) NO radicals.

Four methods were utilized to analyze the free scavenging activity of TTEP. ABTS could be easily converted into radical cation by oxidation reaction with a sensitive color change; thus, the ABTS assay is widely accepted to evaluate the total antioxidant power of compounds [24]. DPPH is well-known as a radical and also a predator for other radicals [30]. O_2_^−^ and NO radical are main forms of ROS and RNS, respectively [31]. The results on ABTS^+^, DPPH, O_2_^−^, and NO scavenging activity of TTEP are shown in Figure 2, with the IC50 of 33.75±4.12, 41.46±4.28, 37.30±5.11, and 23.49±4.34 *μ*g/mL, respectively, suggesting the strong free radical scavenging activity of TTEP.

### 3.3. TTEP Improved the Immune Function in BCG/LPS-Infected Mice

In the acute toxicity study, the mice were orally treated twice a day with 12 h interval for 3 days at a dose of 1.0 g/Kg, equal to about 50.0 g* E. Konishii *pericarp/Kg. No mortality was observed in the coming two weeks. Body weight, feeding, excretion, behavior, and state of consciousness were normal without obvious toxic reaction. All mice were executed and dissected, and no evident histological abnormalities were noticed in the liver, heart, lung, and kidney tissues at the end of animal experiment (data not shown).

**Figure 3:**
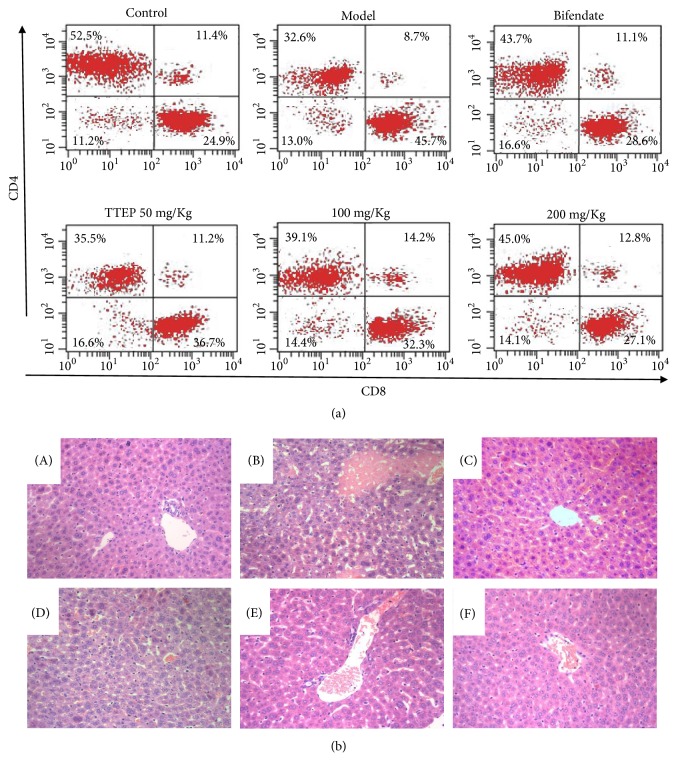
*TTEP attenuated BCG/LPS-induced liver histopathologic architecture*. (a) Flow cytometric analysis of T lymphocyte subgroups of CD3^+^, CD4^+^, and CD8^+^. After the erythrocyte lysis, the anticoagulated blood was incubated CD3^+^, CD4^+^, and CD8^+^ antibody (2:1:1) and analyzed with flow cytometry. (b) Mice liver tissues were detected by H&E staining (200×) after being administered with vehicle (control and BCG/LPS model groups), Bifendate (200 mg/Kg), or TTEP (50, 100, 200 mg/Kg) for 10 days. (A) Control group; (B) BCG/LPS-induced model group; (C) 200 mg/Kg Bifendate group; (D) 50 mg/kg TTEP group; (E) 100 mg/kg TTEP group; (F) 200 mg/kg TTEP group.

Liver, thymus, and spleen are important organs in mammals which are involved in the immune response [32–34]. Therefore, the levels of LI, TI, and SI were firstly measured to analyze the growth and development of immune system (shown in Table 2). In contrast with the control group, the LI, TI, and SI in the BCG/LPS-induced model group were obviously increased (*P*<0.01), indicating severe immune system damage. While the TTEP administration (50, 100 and 200 mg/Kg) gradually reduced the levels of LI, TI, and SI, suggesting that TTEP have immunoregulatory activity in the BCG/LPS-induced mice. The subsets of T cells in the blood were then analyzed to evaluate the whole mice immune function. In contrasted with control group, the CD3^+^ and CD4^+^ were dramatically decreased, while the CD8^+^ was increased in the model group (*P*<0.01), leading to the significant decline in the ratio of CD4^+^/CD8^+^, which represented the serious immune damage induced by the BCG/LPS attack. The frequency of CD3^+^, CD4^+^, CD8^+^ and the ratio of CD4^+^/CD8^+^ were gradually approaching to the control group in the TTEP treatment groups, suggesting that TTEP could improve immune function of BCG/LPS-induced mice (Figure 3(a) and Table 3).

The ALT, AST, and ALP activities were usually determined to assess the liver function. As shown in Table 4, the ALT, AST, and ALP serum levels were dramatically higher in the model group compared with that of the control group (*P*<0.01), indicating significantly hepatic cell damage. However, the ALT, AST, and ALP levels were remarkably reduced with a dose-dependent TTEP treatment (50, 100, and 200 mg/Kg), reflecting the protective effect of TTEP on liver function. HE staining was also adopted to assess the effect of TTEP on the BCG/LPS-infected liver. There were severe pathological changes in the liver lobules of the model group, which is characterized with extensive infiltration of inflammatory cells arranged around the necrosis tissues. Nevertheless, the extent of necrosis was gradually relieved and the inflammatory infiltration was alleviated with the increase of TTEP dosage (Figure 3(b)). All these results showed that TTEP has a protective effect on the immunological hepatoma injury.

### 3.4. TTEP Alleviated Inflammation in BCG/LPS-Infected Hepatoma Injury

**Figure 4:**
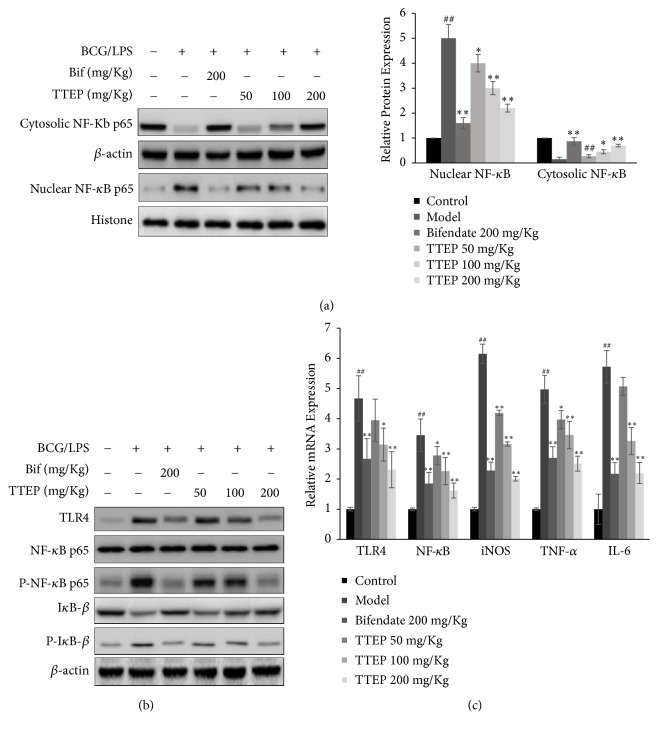
*TTEP alleviated inflammation in BCG/LPS-induced liver injury.* (a) An equal amount (50 *μ*g) of total liver homogenate proteins from each group was detected with *β*-actin as a control. Western blot analysis results show the effect of TTEP on the protein levels of TLR4, NF-*κ*B p65, Phospho-NF-*κ*B p65, I*κ*B-*β*, and Phospho-I*κ*B-*β*. (b) The nuclear and cytosolic fraction of liver homogenate were separated, and then detected by 8-15% SDS-PAGE. The protein expressions of nuclear NF-*κ*B p65 was detected using Histone as the control, while the cytosolic NF-*κ*B p65 levels were measured with *β*-actin as the control. (c) Total RNA of liver tissue extracted with TRIzol reagent and then transcribed reversely into cDNA. Quantitative real-time PCR analysis results show the effect of TTEP on the mRNA levels of TLR4, NF-*κ*B, iNOS, TNF-*α*, and IL-6 of each group. Results are presented as means ± SD of three independent experiments, ^*∗*^*P* < 0.05, ^*∗∗*^*P* < 0.01 compared with the model group; ^##^*P*<0.01 compared with the control group.

To demonstrate the anti-inflammatory mechanism of TTEP in the liver damage, we analyzed the TLR4/NF-*κ*B signaling by western blot analysis (shown in Figures 4(a) and 4(b)). Although the protein levels of NF-*κ*B p65 fraction (referred to as p65) did not show significant change, the expression of TLR4 and the phosphorylated NF-*κ*B p65 subunit (P-p65) were greatly increased, and the expression of the inhibition protein of NF-*κ*B (I*κ*B) was remarkably decreased with the rising concentration of phosphorous I*κ*B*α* (P-I*κ*B*α*) in the model group in contrast with the normal mice (Figure 4(b)). Nevertheless, the treatment of TTEP suppressed the rise of TLR4, P-p65, and P-I*κ*B*α* and upregulated the contents of I*κ*B*α*, which prevent the activation of TLR4/NF-*κ*B signaling. The elevated mRNA expressions of TLR4 and NF-*κ*B induced by BCG/LPS were also downregulated by TTEP administration (shown in Figure 4(c)). Furthermore, we also found that TTEP administration downregulated the magnified nuclear p65 expression induced by BCG/LPS, indicating that TTEP inhibited the translocation of p65 from cytosol into the nucleus (shown in Figure 4(a)). The cytokines and chemokines in liver were also detected to demonstrate the effect of TTEP on the hepatic inflammation. As shown in Table 5, BCG/LPS infection significantly enhanced the protein expression of TNF-*α* and IL-6, as well as the secretion of NO (*P*<0.05 or* P*<0.01), and TTEP administration downregulated these proinflammatory biomarkers in the liver of BCG/LPS-induced mice (*P*<0.05 or* P*<0.01). Besides, TTEP administration (50, 100, 200 mg/Kg) gradually reduced BCG/LPS-induced mRNA transcription of iNOS, TNF-*α*, and IL-6 (shown in Figure 4(c)). These results showed that TTEP regulated the TLR/NF-*κ*B pathway, prevented NF-*κ*B translocate into nucleus, and thus inhibited the expression of the inflammatory cytokines and chemokines.

### 3.5. TTEP Attenuated Oxidative Stress in BCG/LPS-Infected Hepatoma Injury

**Figure 5:**
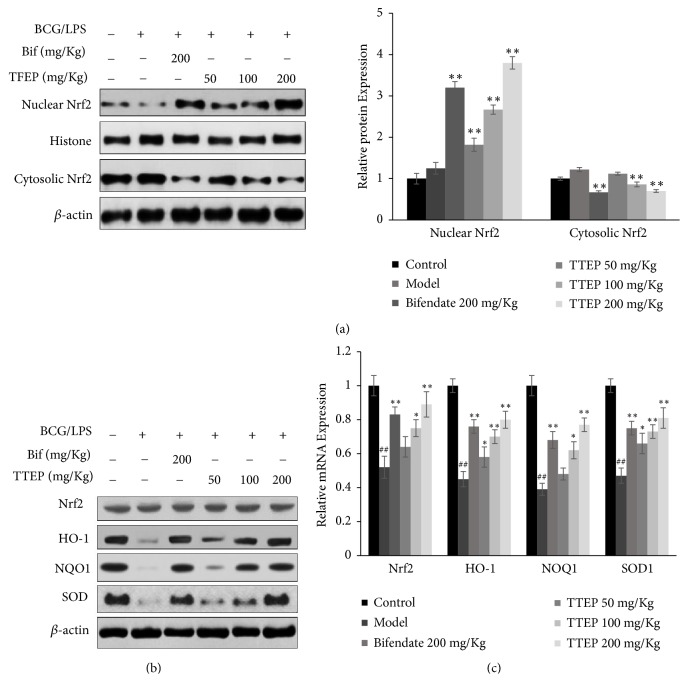
*TTEP ameliorated oxidative stress in BCG/LPS-induced liver injury*. (a) Western blot analysis results show the effect of TTEP on the protein levels of Nrf2, HO-1, NQO1, and SOD of each group. (b) The effect of TTEP on the protein expression of nuclear and cytosolic Nrf2 was tested by western blot. The expressions of nuclear Nrf2 were detected using Histone as the control, while the cytosolic Nrf2 levels were measured with *β*-actin as the control. (c) Quantitative real-time PCR analysis results show the effect of TTEP on the mRNA levels of Nrf2, HO-1, NOQ1, and SOD in each group. Results are presented as means ± SD of three independent experiments, ^*∗*^*P* < 0.05, ^*∗∗*^*P* < 0.01 compared with the model group; ^#^*P*<0.01 compared with the control group.

The antioxidative effect of TTEP has been assessed by the expression of Nrf2/HO-1 pathway (shown in Figure 5). Although no significant changes had been observed in the content of total Nrf2, HO-1, NQO1, and SOD levels were dramatically decreased in the BCG/LPS-induced liver, but could be reversed markedly by TTEP in a dose-dependent manner (Figure 5(b)). In addition, TTEP administration increased the nuclear Nrf2 expression which was suppressed by the BCG/LPS infection (shown in Figure 5(a)). Similarly, the mRNA expression of Nrf2, HO-1, NQO1, and SOD was inhibited by BCG/LPS induction, but upregulated by TTEP treatment in a dose-dependent manner (Figure 5(c)). Furthermore, the antioxidative enzymes SOD, CAT, and GSH-Px as well as the lipid oxidation production MDA were also detected in Table 6. The results showed that BCG/LPS reduced the activity of GSH-Px, CAT, and SOD and elevated MDA content compared with the normal mice. However, TTEP increased the above antioxidative enzymes and thus decreased the content of lipid oxidation production MDA. These data suggested that TTEP facilitated the activation of Nrf2/HO-1 signaling and stimulated the expression of antioxidant and detoxification enzymes.

### 3.6. TTEP Suppressed Apoptosis in BCG/LPS-Infected Hepatoma Injury

**Figure 6:**
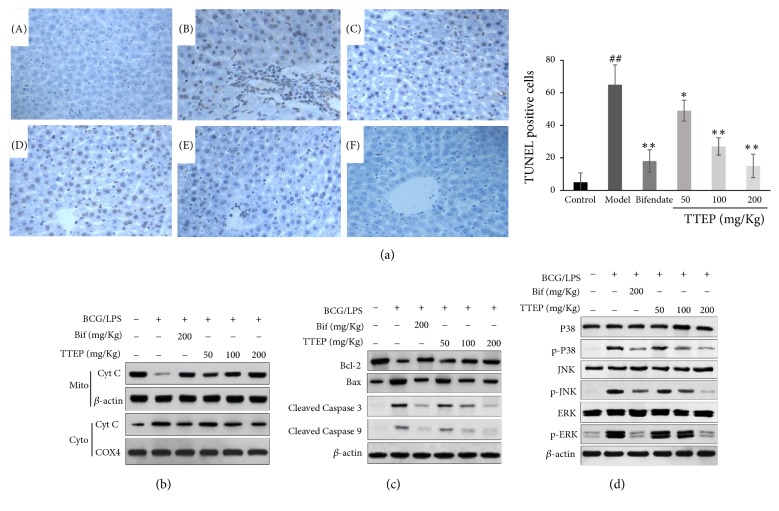
*TTEP suppressed apoptosis in BCG/LPS-induced liver injury*. (a) Representative TUNEL assay in liver sections (200×). (A) Control group; (B) BCG/LPS-induced model group; (C) 200 mg/Kg Bifendate group; (D) 50 mg/kg TTEP group; (E) 100 mg/kg TTEP group; (F) 200 mg/kg TTEP group. (b) The mitochondria of liver tissues were extracted from the liver homogenate. The mitochondrial and cytosolic protein expressions of Cyt C were detected using Cytochrome C oxidase subunit IV (COX4) as the control, while the cytosolic Cyt C levels were measured with *β*-actin as the control. (c) The expression of major mitochondrial apoptosis proteins Bcl-2, Bax, cleaved caspase-3 and 9 was detected by western blot using *β*-actin as the control. (d) Western blot analysis results show the effect of TTEP on the protein levels of P38, Phospho-P38, JNK, Phospho-JNK, ERK and Phospho-ERK with the control of *β*-actin.

The effect of TTEP on the BCG/LPS-induced cell death was also measured by TUNEL staining. As shown in Figure 6(a), TUNEL positive cells were remarkably increased in the BCG/LPS-infected mouse compared with that of normal mice but showed a dose-dependent inhibition by TTEP administration.

The release of Cyt C from mitochondria into cytosol is key events in cell apoptosis [35]. Hence, we measured the levels of Cyt C in both nucleus and cytosol. Remarkable rise of Cyt C was observed in the cytosolic fraction by BCG/LPS induction, while a dose-dependent inhibition of Cyt C release could be found after TTEP treatment (shown in Figure 6(b)). Cytosolic Cyt C activates the cell apoptosis pathways including caspase and Bcl-2 families, leading to the programmed cell death [16, 17]. The expression of proapoptotic Bcl-2 decreased and that of antiapoptotic Bax increased in the BCG/LPS-infected hepatocyte. However, treating with ascending TTEP dosage gradually upregulated Bcl-2 concentrations and downregulated Bax levels (shown in Figure 6(c)). The levels of apoptosis activator Caspase 9 and apoptosis executioner Caspase 3 were also determined in Figure 6(c).

MAPK signaling is closely involved in the regulation of the mitochondrial permeability-mediated activation of apoptotic cascade [36]. Compared with the control group, the BCG/LPS-infected model group showed a sharply increase in the expressions of cleaved Caspase 3 and 9. However, TTEP administration suppressed the induced Caspase-3 and 9 activation by regulating the MAPK signaling (shown in Figure 6(d)). Contrary to the control group, the levels of phosphorylated form of p38, JNK, and ERK dramatically increased in the BCG/LPS-infected mice. However, treatment with TTEP inhibited the raised phosphorylation of p38, JNK, and ERK. These results indicated that TTEP could alleviate the BCG/LPS-inducted hepatocytes apoptosis by modulating the MAPK signal and preventing apoptosis pathways.

## 4. Discussion

LPS-induced liver injury has been recognized as the pathological basis of viral hepatic diseases, which has become the primary cause of hepatogenic morbidity and mortality all over the world [4]. The pericarp of* E. konishii* is usually used in cooking soup as a detumescent and analgesic agent to enhance liver function in South China [21, 22]. Previous studies show that* E. konishii* pericarp has potent anti-inflammation, antihepatoma, and analgesia abilities with high triterpenes [23, 37]. Hence, we investigate the chemical constituent and hepatoprotective activities of the total triterpenes of the* E. konishii* pericarp (TTEP) using BCG/LPS-infected mice model.

Eight pentacyclic triterpenoid acids were separated from TTEP and identified by 1D, 2D-NMR, and MS (shown in Figure 1 and Supplementary Materials). Pentacyclic Triterpenes have been reported to be promising anticancer drugs with potent anti-inflammatory, antioxidative, antiangiogenic activities [38–40]. No surprisingly, the* in vitro* free radical scavenging assays showed that TTEP could significantly scavenge free radicals (ABTS^+^, DPPH, O_2_^−^, and NO), indicating the strong free radical scavenging activity of TTEP (shown in Figure 2).

No mortality or toxic signs were observed at the maximal dose that is equal to 50.0 g* E. Konishii *pericarp/Kg, implying that TTEP has a favorable toxicity profile.

The immunological liver injury induced by BCG/LPS has been accepted to reflect the accurate clinical situation of viral fulminant hepatitis [5–7]. In this study, the elevated levels of LI, TI, SI, ALT, AST, and ALP (Tables 2 and 3) and the disproportion of T cell subset as well as the large necrosis and inflammatory infiltration in the liver lobule (Figure 3(b)) are remarkable characters of BCG/LPS-induced hepatic damage. CD3^+^ T lymphocytes level represents the integral cellular immunity, while CD4^+^ and CD8^+^ T lymphocytes represent helper and killer T lymphocytes, respectively. The administration of TTEP increased the frequency of CD3^+^, CD4^+^ and CD4^+^/CD8^+^ (Figure 3(a) and Table 3) and inhibited the induced levels of LI, TI, SI, ALT, AST, and ALP (Tables 2 and 4) as well as alleviated the hepatocyte damage (Figure 3(b)), indicating the significant protective effect on immunological liver injury.

LPS is a potent stimuli of inflammatory response and is also vital for the oxidative/nitrosative stress [41]. TLR4/NF-*κ*B signaling plays a crucial role in the LPS-associated inflammatory responses [42]. LPS binding protein (LBP) recognizes and binds LPS to activate the transmembrane receptor TLR4, which would initiate the downstream NF-*κ*B signaling [43]. Normally, NF-*κ*B exists as an inactive complex with I*κ*B in the cytosol [44]. Upon stimulation, I*κ*B is phosphorylated and then ubiquitinated by 26S proteasome, which facilitates free NF-*κ*B p65 subunit into the nucleus [45]. Then the nuclear p65 regulates the expression of genes associated with the inflammatory response, cell differentiation, and apoptosis [46]. As expected, our study showed that BCG/LPS could stimulate the inflammation, oxidant and nitrogen-sensitive NF-*κ*B signaling and significantly increase the NO production and iNOS expression, as well as TNF-*α* and IL-6 cytokine levels in mice liver. However, the elevation of these chemokines proinflammatory and cytokines could be dose-dependently attenuated by TTEP (Figure 4 and Table 5).

Oxidative stress is a key factor in the pathogenesis of liver disease and the peroxidation of protein and lipid are common events in hepatic toxicity [47]. MDA, a marker of lipid and protein oxidation [48], was remarkably increased in mice liver by BCG/LPS infection but could be significantly reduced by TTEP treatment (Table 6). Nrf2 has been recognized as one of the master regulators against oxidative injury by alleviating the inflammatory response [49, 50]. Under normal conditions, Nrf2 interacts with the Keap1 in the cytosol and is rapidly degraded by the ubiquitin-proteasome pathway. Contrarily, with oxidative stress stimulation, Nrf2 separated from the complex and translocated into the nucleus to activate the antioxidant genes, such as NQO1 and HO-1 [14, 51]. The Nrf2/HO-1 activation could also promote the expression of endogenous enzymes, including glutathione (GSH) and SOD [50]. TTEP treatment activated Nrf2/HO-1 signaling to promote the downstream expression of antioxidant and detoxifying genes, which greatly suppressed the oxidative damage induced by BCG/LPS (Figure 5). In addition, recent reports have also shown that activation of Nrf2/HO-1 pathways could attenuate NF-*κ*B signaling, along with the downregulation of the proinflammatory cytokines and chemokines [52].

The excessive ROS and inflammation filtration induced by BCG/LPS results in significant damage to hepatocyte structures, which eventually leads to liver demise [5–7]. As mitochondria is the key organelle to produce ATP through oxidative phosphorylation, mitochondrial dysfunction is greatly involved in the cell apoptosis [53, 54]. The release of Cyt C is from the intermitochondrial space into the cytoplasm when the mitochondrial membrane permeability is altered. Cytosolic Cyt C then binds to Caspase 9 and apoptosis protease Apaf-1 and subsequently activates the executioner Caspase 3 during the programmed cell death [16, 17]. In addition, MAPK phosphorylation controls the balance of pro- and antiapoptotic proteins [36]. In this study, the hepatic apoptosis induced by BCG/LPS was verified by the increased TUNEL positive cells in the BCG/LPS-infected liver, imbalance of Bcl2/Bax, elevated level of cytosolic Cyt C, and activation of both MAPK and Caspase pathways (Figure 6). However, TTEP administration effectively decreased the level of cytosolic Cyt C, proapoptotic Bcl-2, cleaved Caspase 3 and 9, increased the content of antiapoptotic Bax, and inhibited the activation of MAPK pathways, indicating that TTEP could suppress BCG/LPS-induced hepatocyte apoptosis.

## 5. Conclusions

**Figure 7:**
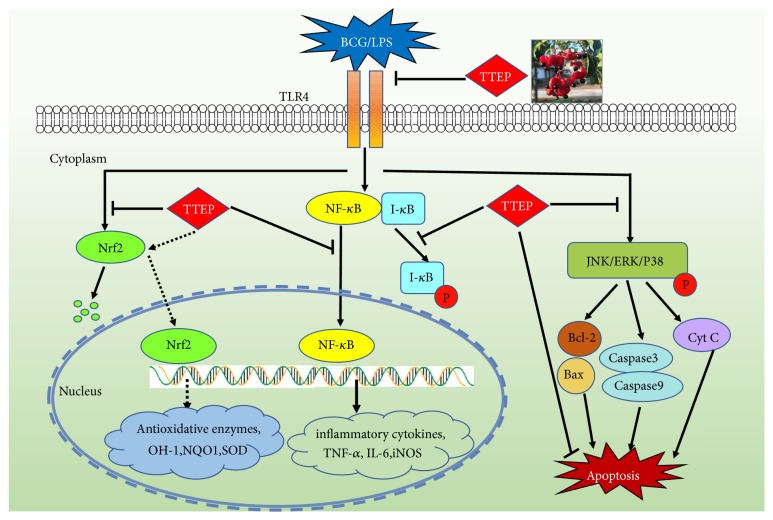
Schematic representation of the mechanisms of total triterpenes of the E. konishii pericarp (TTEP) in attenuating BCG/LPS-induced immunological liver injury through suppressing TLR4/NF-*κ*B inflammatory signaling, promoting the expression of antioxidative Nrf2/HO-1 pathway, and inhibiting MAPK-associated hepatocyte apoptosis signaling.

The total triterpenes of* Euscaphis konishii* Hayata pericarp (TTEP) showed the immune boosting effect and hepatic protective activity against inflammation, oxidative stress, and apoptosis in the BCG/LPS-induced liver injury. The results from the present study indicated that TTEP strengthened the immunity of BCG/LPS-infected mice, ameliorated biochemical and histological alteration, suppressed the TLR4/NF-*κ*B inflammatory signaling, promoted the expression of antioxidative Nrf2 and PPAR pathways, and inhibited the hepatocyte apoptosis by suppressing MAPK-associated signaling (schematic representation of the mechanisms shown in Figure 7). These findings suggest that the pericarp of* E. konishii* might be a promising natural food for immunological hepatic injury.

## Figures and Tables

**Table 1:** List of primers in PCR amplification.

Gene	Accession No.	Primer Sequence	Amplicon Size (bp)
TLR4	NM_021297.3	Forward: 5'-TAGCCATTGCTGCCAACATC-3' Reverse: 5'- CCTCAGCAGGGACTTCTCAA -3'	194
NF-*κ*B	AY521463.1	Forward: 5'- GGAGACATCCTTCCGCAAAC -3' Reverse: 5'- AGGTCCTTCCTGCCCATAAC -3'	103
iNOS	M87039.1	Forward: 5'- ATGCGAAAGGTCATGGCTTC-3' Reverse: 5'- CCCAAATGTGCTTGTCACCA -3'	198
TNF-*α*	D84199.2	Forward: 5'- CCCAAAGGGATGAGGTGAGT-3' Reverse: 5'- GGTCTGGGCCATAGAACTGA -3'	185
IL-6	M24221.1	Forward: 5'- ACTTCACAAGTCCGGAGAGG-3' Reverse: 5'-TGCAAGTGCATCATCGTTGT -3'	174
Nrf2	BC026943	Forward: 5'-TGGCTGATACTACCGCTGTT-3' Reverse: 5'-TGGAGAGGATGCTGCTGAAA-3'	162
HO-1	NM_010442.2	Forward:5'-TGTCTGAGGCCTTGAAGGAG-3' Reverse: 5'-CAGGGCCGTGTAGATATGGT-3'	146
NQO1	BC004579	Forward: 5'-ACAGGTGAGCTGAAGGACTC-3' Reverse: 5'-CCAAACCACTGCAATGGGAA-3'	155
SOD1	NM_011434.2	Forward: 5'-TCCACGTCCATCAGTATGGG-3' Reverse: 5'- AATGGACACATTGGCCACAC -3'	164
*β*-actin	M12481.1	Forward: 5'-CCAGCCTTCCTTCTTGGGTA-3' Reverse: 5'-CAATGCCTGGGTACATGGTG-3'	143

Full-length gene sequences were obtained from the Nucleotide database (www.ncbi.nlm.nih.gov/nuccore/), and primers were designed using primer premier 5.0 software.

**Table 2:** Effect of TTFP on LI, TI, and SI of mice induced by BCG/LPS ($\bar{x}\pm s$, n=10).

Group	LI (mg/g)	SI (mg/g)	TI (mg/g)
Control	83.53 *± *12.48	34.41 *± *5.87	26.95 *± *3.76
Model	179.83 *± *37.65^##^	71.93 *±* 11.82^##^	53.70 *± *7.69^##^
Bifendate	112.66 *± *28.49^*∗∗*^	45.30 *± *6.31^*∗∗*^	34.67 *±* 4.23^*∗∗*^
TTEP 50 mg/Kg	160.58 *± *36.47	64.78 *± *9.55^*∗*^	49.74 *± *5.76
100 mg/Kg	137.46 *± *28.09^*∗*^	53.14 *± *6.57^*∗*^	40.32 *± *5.67^*∗*^
200 mg/Kg	108.48 *±* 27.32^*∗∗*^	47.86 *±* 6.06^*∗∗*^	32.16 *± *4.58^*∗∗*^

LI, SI, and TI represent Liver Index, Spleen Index, and Thymus Index, respectively. LI = [liver weight (mg)/body weight (g)]; SI = [spleen weight (mg)/body weight (g)]; TI = [thymus weight (mg)/body weight (g)]. ^*∗*^*P* < 0.05, ^*∗∗*^*P* < 0.01 compared with the model group; ^#^*P*<0.05, ^##^*P*<0.01 compared with the control group.

**Table 3:** Effect of TTEP on the T lymphocyte subgroups of BCG/LPS induced live injury ($\bar{x}\pm s$, n=10).

Group	CD3^+^ (%)	CD4^+^ (%)	CD8^+^ (%)	CD4^+^ / CD8^+^ (%)
Control	76.1 *± *6.4	52.5 *± *6.0	24.9 *± *6.8	2.11 *±* 0.42
Model	38.6 *± *4.2^##^	32.6 *± *5.8^##^	45.7 *± *7.9^#^	0.71 *±* 0.08^##^
Bifendate	62.3 *± *7.5^*∗∗*^	43.7 *± *8.1^*∗∗*^	28.6 *± *7.3^*∗*^	1.52 *±* 0.41^*∗∗*^
TFEP 50 mg/Kg	43.5 *± *5.6	35.5 *± *5.1	36.7 *± *8.3	0.96 *±* 0.17^*∗*^
100 mg/Kg	56.4 *±* 7.3^*∗*^	39.1 *±* 6.3^*∗*^	32.3 *± *6.7^*∗*^	1.21 *±* 0.36^*∗∗*^
200 mg/Kg	67.7 ± 5.4^*∗∗*^	45.0 ± 7.4^*∗∗*^	27.1 ± 5.8^*∗*^	1.66 ± 0.49^*∗∗*^

Anticoagulated blood was treated with erythrocyte lysate to get the lymphocyte suspension and then incubated with CD3+, CD4+, and CD8+ antibody before analysis with flow cytometry. ^*∗*^*P* < 0.05, ^*∗∗*^*P* < 0.01 compared with the model group; ^#^*P*<0.05, ^##^*P*<0.01 compared with the control group.

**Table 4:** Effect of TTEP on serum levels of ALT, AST, and ALP ($\bar{x}\pm s$, n=10).

Group	ALT (U/L)	AST (U/L)	ALP (U/L)
Control	36.2 *± *9.0	43.0 *± *16.8	87.6 *± *10.4
Model	158.2 *± *32.3^##^	163.7 *± *13.6^##^	162.6 *± *16.9^##^
Bifendate	95.0 *± *16.9^*∗∗*^	105.7 *± *8.1^*∗∗*^	115.6 *±* 12.3^*∗∗*^
TTEP 50 mg/Kg	123.6 *± *20.7^*∗*^	122.5 *± *15.1^*∗*^	143.7 *± *13.6
100 mg/Kg	105.2 *±* 20.1^*∗∗*^	105.1 *±* 10.3^*∗∗*^	132.3 *± *9.7^*∗∗*^
200 mg/Kg	82.4 *±* 19.0^*∗∗*^	79.8 *± *10.6^*∗∗*^	112.1 *±* 15.8^*∗∗*^

Mouse blood samples were centrifuged to get the serum, and alanine aminotransferase (ALT), aspartate transaminase (AST), and alkaline phosphatase (ALP) activities were detected by colorimetric method. ^*∗*^*P* < 0.05, ^*∗∗*^*P* < 0.01 compared with the model group; #P<0.05, ##P<0.01 compared with the control group.

**Table 5:** Effect of TTFP on TNF-*α*, IL-6, and NO of immunological liver injury mice ($\bar{x}\pm s$, n=10).

Group	TNF-*α* (pg/mL)	IL-6 (pg/mL)	NO (*μ*mol/gprot)
Control	63.4 *± *16.8	37.3 *± *8.4	1.47 *± *0.36
Model	39.7 *± *11.0^##^	143.6 *± *26.5^##^	3.62 *± *0.64^#^
Bifendate	55.6 *± *12.4^*∗∗*^	66.1 *± *13.3^*∗*^	1.94 *±* 0.47^*∗∗*^
TTEP 50 mg/Kg	42.4 *± *13.1	102.4 *± *19.7^*∗*^	2.85 *± *0.43^*∗*^
100 mg/Kg	50.7 *± *14.3^*∗*^	87.6 *± *15.2^*∗∗*^	2.30 *± *0.52^*∗∗*^
200 mg/Kg	58.3 *± *12.9^*∗∗*^	55.7 *± *10.7^*∗∗*^	1.91 *±*.051^*∗∗*^

Mouse liver samples were homogenized with icy saline and centrifuged to get the supernatants. The levels of TNF-*α* and IL-6 were detected with ELISA kits, while NO contents were detected by Griess Reagent Assay. ^*∗*^*P* < 0.05, ^*∗∗*^*P* < 0.01 compared with the model group; ^#^*P*<0.05, ^##^*P*<0.01 compared with the control group.

**Table 6:** Effect of TEF on the SOD, GSH, CAT, and MDA activities in liver ($\bar{x}\pm s$, n=10).

Group	SOD (U/mg protein)	GSH (U/mg protein)	CAT (U/mg protein)	MDA (nmol/mg protein)
Control	83.1 *± *6.4	52.5 *± *6.0	64.8 *± *8.2	24.9 *± *6.8
Model	35.6 *± *4.2^##^	24.7 *± *5.8^##^	43.0 *± *7.2^#^	42.6 *± *7.9^##^
Bifendate	62.3 *± *7.5^*∗∗*^	40.7 *± *8.1^*∗∗*^	54.2 *± *6.6^*∗*^	30.6 *± *7.3^*∗∗*^
TTEP 50 mg/Kg	43.5 *± *5.6^*∗*^	32.5 *± *5.1^*∗*^	50.5 *± *7.8	38.7 *± *8.3
100 mg/Kg	56.4 *±* 7.3^*∗*^	39.1 *±* 6.3^*∗∗*^	52.9 *± *7.4^*∗*^	32.3 *± *6.7^*∗*^
200 mg/Kg	66.7 ± 8.4^*∗∗*^	43.8 ± 7.4^*∗∗*^	58.1 ± 5.8^*∗*^	28.1 ± 5.8^*∗∗*^

Mouse liver samples were homogenized with icy saline and centrifuged to get the supernatants and the concentrations of SOD, GSH, and CAT and MDA were evaluated using commercial diagnostic kits. ^*∗*^*P* < 0.05, ^*∗∗*^*P* < 0.01 compared with the model group; ^#^*P*<0.05, ^##^*P*<0.01 compared with the control group.

## Data Availability

The data used to support the findings of this study are included within the article.

## Supplementary Materials

Supplementary Materials Supplementary Materials provide the NMR data of 8 triterpenes isolated from the* E. konishii* pericarp mentioned in Section 3.1.
