# Supplementary material for: Protective Effect of the Total Triterpenes of Euscaphis konishii Hayata Pericarp on Bacillus Calmette-Guérin Plus Lipopolysaccharide-Induced Liver Injury
Source: Evid Based Complement Alternat Med. 2019 Apr 4;2019:1806021. doi: 10.1155/2019/1806021 (PMC6475556; doi:10.1155/2019/1806021)
Supplement: Supplementary Materials — provide the NMR data of 8 triterpenes isolated from the E. konishii pericarp mentioned in Section 3.1. [file 1806021.f1.docx]

**Supplementary Materials**

**NMR Data of TTEP**

Compound **1**, Betulinic acid, white powder, ESI-MS m/z: 457.3 [M+H]*^+^*, C_30_H_48_O_3_, ^1^H-NMR (pyridine-*d5*, 500 MHz) δ: 4.97 (1H, brs, H-29α), 4.79 (1H , brs, H-29β), 1.82 (3H, s, H-23), 1.25 (3H, s, H-24), 1.09 (3H, s, H-30), 1.08 (3H, s, H-26), 1.01 (3H, s, H-25), 0.85 (3H, s, H-27); ^13^C-NMR (pyridine-*d5*, 125 MHz) δ: 179.3 (C-28), 151.8 (C-20), 110.4 (C-29), 78.6 (C-3), 57.1 (C-17), 56.4 (C-5), 51.4 (C-9), 50.2 (C-19), 48.25 (C-18), 43.32 (C-14), 41.6 (C-8), 40.0 (C-4), 39.8 (C-13), 39.1 (C-1), 38.1 (C-10), 38.0 (C-22), 35.3 (C-7), 33.4 (C-16), 31.7 (C-21), 30.8 (C-15), 29.1 (C-23), 28.8 (C-2), 26.6 (C-12), 21.7 (C-11), 20.0 (C-30), 19.0 (C-6), 17.0 (C-24 , 26), 16.8 (C-25),15.4 (C-27).

Compound **2**, Oleanolic acid, white powder, EI-MS m /z: 456.8 [M]^+^, C_30_H_48_O_3_. ^1^H- NMR (CDCl_3_, 500 MHz) δ: 5.21 (1H, s, H-12), 1.14 (3H, s, H-23), 1.01 (3H, s, H-29), 0.89 (3H, s, H-30), 0.84 (3H, s, H-27), 0.75 (3H, s, H-26), 0.70 (3H, s, H-24), 0.65 (3H, s, H-25); ^13^C NMR (CDCl_3_, 125 MHz) δ: 183.4 (C-28), 143.1 (C-13), 121.9 (C-12), 78.4 (C-3), 55.0 (C-5), 46.5 (C-9), 45.3 (C-19), 44.5(C-17), 41.0(C-14), 40.0 (C-18), 39.0 (C-1), 38.2 (C-8), 37.9 (C-4), 37.1 (C-10), 32.6 ( C-29), 32.3 (C-21), 31.4 (C-7), 31.0 (C -22), 30.2 (C-23), 29.0 (C-20), 28.0 (C- 15), 26.5 (C-2), 26.0(C-27), 24.5(C-16), 22.4 (C-11), 22.2 (C-30),19.0 (C-6), 16.1 (C-24), 15.8 (C-26), 14.7 (C-25).

Compound **3**, Siaresinolic acid, white powder, ESI-MS m/z: 472.8 [M]^+^, [C_30_H_48_O_4_](https://pubchem.ncbi.nlm.nih.gov/search/#collection=compounds&query_type=mf&query=C30H48O4&sort=mw&sort_dir=asc).^1^H-NMR (pyridine-*d5*, 500 MHz) δ: 5.57 (1H, brs, H-12), 3.63 (2H, brs, H-18, 19), 3.43 (1H, dd, *J* =11.1, 4.8 Hz, H-3), 1.65 (3H, s, H-27), 1.23 (3H, s, H-23), 1.18 (3H, s, H-29), 1.11 (3H, s, H-30), 1.07 (3H, s, H-26), 1.02 (3H, s, H-24), 0.92 (3H, s, H-25); ^13^C-NMR (125 MHz, pyridine-*d5*) δ: 180. 7 (C-28), 144.9 (C-13), 122. 8 (C-12), 81.1 (C-19 ), 78.0 (C-3 ), 55.7 (C-5 ), 48.2 (C-9 ), 45.9 (C-17), 44.6 (C-18 ), 42.2 (C-14), 40.2 (C-8 ), 39.8 (C-4 ), 39.2 (C-1), 37.3 (C-10), 35.5 (C-20 ), 33.4 (C-7), 33.2 (C-22), 29.0 (C-23 ), 29.0 (C-29), 28.6 (C-15), 28.6 (C-21 ), 28.2 (C-2 ), 27.9 (C-16), 24.6 (C-27), 24.5 (C-30), 24.0 ( C-11), 18.7 ( C-6 ), 17.3 (C-26), 16.2 (C-24), 15.2 ( C-25).

Compound **4**, ursolic acid, white powder, EI-MS m/z: 457.4 [M+H]^+^, C_30_H_48_O_3_. ^1^H NMR (CDCl_3_,500 MHz) δ: 5.25 (1H, s, H-12), 3.15 (1H, m, H-3), 0.97 (3H, s, H-26), 0.91 (3H, s, H-27), 0.88 (3H, s, H-25), 0.80 (3H, s, H-24), 0.77 (3H, d, *J* = 3.3Hz, H-29), 0.74 (3H, s, H-23), 0.71 (3H, d, *J* = 3.3 Hz, H-30); ^13^C NMR (CDCl_3_, 125 MHz) δ: 207.0 (C-28), 136.19 (C-13), 126.0 (C-12), 78.7 (C-3), 54.0 (C-5), 51.6 (C-18), 50.0 (C-17), 47.0 (C-9), 43.0 (C-14), 39.0 (C-4), 38.4 (C-8), 37.5 (C-1), 37.5 (C-19), 37.5 (C-20), 35.1 (C-10), 32.0 (C-7), 32.0 (C-22), 30.1 (C-15), 29.5 (C-16), 28.5 (C-23), 27.0 (C-21), 26.0 (C-2), 23.5 (C-11), 22.2 (C-27), 20.1 (C-30), 17.1 (C-6), 16.5(C-25), 15.8 (C-29),15.1 (C-26),14.3 (C-24).

Compound **5**, Pomolic acid, white powder, EI-MS m/z: 472.4 [M]^+^, C_30_H_48_O_5_. ^1^H NMR (pyridine-*d5*, 600MHz) δ: 0.92 (3H, s, H-25), 1.04 (3H, s, H-24), 1.14 (3H, s, 26-H), 1.14 (3H, d, J=6.0 Hz, H-30), 1.24 (3H, s, H-23), 1.45 (3H, s, H-29), 1.72 (3H, s, H-27), 3.05 (1H, s, H-18), 3.42 (1H, dd, *J*=10.0, 3.9 Hz, H-3α), 5.63 (1H, t, *J*=3.5 Hz,H-12); ^13^C NMR (150 MHz, pyridine-*d5*) δ: 181.4 (C-28), 140.6 (C-13), 128.5 (C-12), 78.8 (C-3), 73.2 (C-19), 56.4 (C-5), 55.2 (C-18), 48.9 (C-17), 48.4 (C-9), 42.8 (C-20), 42.6 (C-14), 40.8 (C-8), 37.8 (C-10), 37.8 (C-22), 34.2 (C-7), 29.9 (C-15), 29.4 (C-24),

28.6 (C-2), 27.6 (C-29), 27.5 (C-21), 27.0 (C-16), 24.8 (C-27), 24.6 (C-11), 19.4 (C-6), 18.0 (C-26), 17.6 (C-23), 17.1 (C-30), 16.8 (C-25).

Compound **6**, Euscaphic acid, white powder, EI-MS m/z: 511.4 [M+Na]^+^, C_30_H_48_O_5_. ^1^H NMR (pyridine-*d5*, 600MHz) δ: 0. 91 (3H, s, H-24), 0.99 (3H, s, H-25),1.12 (3H, s, H-26),1.14 (3H, d, *J*=6.5 Hz, H-30), 1.28 (3H, s, H-23), 1.43 (3H, s, H-29),1.66 (3H, s, H-27), 2.35 (1H, m, H-15β), 3.06 (1H, s, H-18), 3.14 (1H, m, H-16α), 3.77 (1H, d, *J* =2.5 Hz, H-3β), 4.33 (1H, dt, *J*=6.8, 2.7 Hz, H-2β), 5.60 (1H, m, H-12); ^13^C NMR (150 MHz, pyridine-*d5*) δ: 181 (C-28), 140.3 (C-13), 128.3 (C-12), 79.7 (C-3), 73.0 (C-19), 66.5 (C-2), 55.0 (C-18), 49.2 (C-5), 48.7 (C-17), 48.0 (C-9), 43.3 (C-1), 42.8 (C-20), 42.6 (C-14), 41.9 (C-8), 39.2 (C-4), 39.0 (C-10), 38.8 (C-22), 33.9 (C-7), 29.8 (C-23), 29.6 (C-15), 27.4 (C-29), 27.3 (C-21), 26.8 (C-16), 25.1 (C-27), 24.5 (C-11), 22.7 (C-24), 19.0 (C-6), 17.7 (C-26), 17.2 (C-25), 17.1 (C-30).

Compound **7**, Tormentic acid, white powder, EI-MS m/z: 511.4 [M+Na]^+^, C_30_H_48_O_5_. ^1^H NMR (pyridine-*d5*, 600MHz) δ: 0.90 (3H, s, H-25), 1.01 (3H, s, H-24), 1.15 (3H, s, H-26), 1.16 (3H, d, *J*=6.2 Hz, H-30), 1.30 (3 H, s, H-23), 1.45 (3 H, s, H-29),1.67 (3H, s, H-27), 2.35 (1H, m, H-15β), 3.08 (1H, s, H-18), 3.16 (1H, m, H-16α), 3.36 (1H, d, *J* =9.2 Hz, H-3α), 4.15 (1H, dt, *J*=3.8, 9.2 Hz, H-2β), 5.60 (1H, m, H-12); ^13^C NMR (150 MHz, pyridine-*d5*) δ: 181.6 (C-28), 141.2 (C-13), 129.0 (C-12), 84.0 (C-3), 73.6 (C-19), 69.1 (C-2), 56.6 (C-5), 55.5 (C-18), 49.3 (C-17), 48.7 (C-9), 44.5 (C-1), 43.2 (C-20), 43.1 (C-14), 42.5 (C-8), 40.8 (C-4), 39.6 (C-10), 39.0 (C-22), 34.2 (C-7), 30.3 (C-23), 30.1 (C-15), 28.4 (C-29), 28.2 (C-21), 27.2 (C-16), 25.6 (C-27), 25.3 (C-11), 19.6 (C-6), 18.7 (C-24), 18.3 (C-26),17.8 (C-25), 17.3 (C-30).

Compound **8**, Corosolic acid, white powder, EI-MS m/z: 472.4 [M]^+^, C_30_H_48_O_5_. ^1^H NMR (pyridine-*d5*, 600MHz) δ: 0.98 (3H, d, *J*=6.5 Hz, H-29), 1.00 (3H, s, H-25), 1.01 (3H, d, *J*=6.0 Hz, H-30), 1.02 (3H, s, H-24), 1.10 (3H, s, H-26), 1.24 (3H, s, H-27), 1.30 (3H, s, H-23), 2.65 (1H, d, *J*=10.5, 4.0 Hz, H-18), 3.43 (1H, d, *J*=9.7 Hz,

H-3α), 4.12 (1H, dt, *J*=3.8, 9.6 Hz, H-2β), 5.48 (1H, t, *J*= 3.2 Hz, H-12); ^13^C NMR (150 MHz, pyridine-*d5*) δ: 181.2 (C-28), 140.0 (C-13), 125.8 (C-12), 84.1 (C-3), 69.1 (C-2), 56.4 (C-5), 54.1 (C-18), 48.7 (C-9), 48.6 (C-1), 48.3 (C-17), 42.5 (C-14), 40.5 (C-4), 40.4 (C-8), 40.2 (C-19), 40.2 (C-20), 39.0 (C-10), 37.8 (C-22), 34.0 (C-7), 31.5 (C-21), 31.5 (C-16), 29.8 (C-23), 29.1 (C-15), 24.2 (C-27), 24.0 (C-11), 21.8 (C-29), 19.3 (C-6), 18.2 (C-24), 18.0 (C-26), 17.8 (C-30), 17.4 (C-25).
